# Associations of CT‐Derived Skeletal Muscle Index and Density With Postoperative Outcomes Following Colorectal Cancer Surgery

**DOI:** 10.1002/jcsm.70384

**Published:** 2026-09-23

**Authors:** Efthymios Papadopoulos, Brian A. Irving, Guillaume Spielmann, MingDe Lin, Kelly R. Finan

**Affiliations:** ^1^ School of Kinesiology Louisiana State University Baton Rouge Louisiana USA; ^2^ Department of Radiology and Biomedical Imaging Yale University New Haven Connecticut USA; ^3^ Visage Imaging, Inc. San Diego California USA; ^4^ Our Lady of the Lake Medical Center Baton Rouge Louisiana USA

**Keywords:** colorectal cancer, myosteatosis, postoperative outcomes, sarcopenia, skeletal muscle, surgery

## Abstract

**Background:**

Preoperative skeletal muscle index (SMI) and skeletal muscle density (SMD) are gaining attention as predictors of postoperative outcomes following colorectal cancer (CRC) surgery. We examined the associations between computed tomography (CT)‐based SMI and SMD with postoperative outcomes following CRC resection.

**Methods:**

This was a retrospective cohort study of patients who were surgically treated for CRC at a regional hospital in Baton Rouge, Louisiana, United States, from October 2018 to February 2024. CT‐based SMI (cm^2^/m^2^) and SMD (Hounsfield units) at the third lumbar vertebra (L3) were used as continuous and binary predictors in multivariable analyses. Low SMI and low SMD were each defined based on two common definitions. Outcomes included length of stay (LOS), severe postoperative complications, discharge location, 90‐day mortality and overall mortality (OM). The associations of SMI and SMD with LOS were examined using negative binomial regression. Logistic regression was used to examine the associations of SMI and SMD with severe postoperative complications and discharge location. Survival analysis was used to determine the associations of SMI and SMD with 90‐day mortality and OM.

**Results:**

A total of 415 patients (mean age 63.7, SD 13.2) were included in the analysis, of whom 189 (45.5%) were females. The median LOS was 4 days, while 50 (12.0%) of patients experienced at least one severe postoperative complication. A total of 30 (7.2%) patients were discharged to a non‐home location, while 12 (2.9%) patients died within 90 days. Over a median follow‐up of 31.6 months, 75 (18.1%) patients died. In multivariable analyses, each 5‐unit decrease in SMD was associated with increased LOS (incidence rate ratio [IRR] 1.06, 95%CI 1.02–1.10, *p* = 0.003), greater odds of severe postoperative complications (odds ratio [OR] 1.25, 95%CI 1.02–1.53, *p* = 0.029) and non‐home discharge (OR 1.40, 95%CI 1.09–1.79, *p* = 0.007), as well as a higher risk of OM (hazard ratio [HR] 1.26, 95%CI 1.09–1.46, *p* = 0.002). In unadjusted analysis, each 5‐unit decrease in SMD was significantly associated with 90‐day mortality (HR 1.36, 95%CI 1.04–1.78, *p* = 0.024). Low SMD based on two different definitions predicted LOS (IRR 1.21, 95%CI 1.05–1.41, *p* = 0.010; IRR 1.28, 95%CI 1.11–1.48. *p* < 0.001), while only one definition was significantly associated with a higher risk of OM (HR 1.93, 95%CI 1.08–3.43, *p* = 0.027). SMI as a continuous or binary predictor was not significantly associated with postoperative outcomes.

**Conclusions:**

CT‐based SMD rather than SMI appears to be a more relevant predictor of postoperative outcomes following CRC surgery. Future research should explore whether prehabilitation can improve SMD prior to CRC surgery.

## 1 Introduction

Colorectal cancer (CRC) is the third most common cancer among males and females in the United States [1]. Additionally, approximately 55 000 patients will die of CRC in the United States by the end of 2026 [1]. Surgery is the most common primary treatment for CRC [2, 3]. Although surgery improves CRC prognosis, the occurrence of postoperative complications prolongs length of hospital stay (LOS) and increases the risk of morbidity (~35%) and mortality (1%–16.4%) [4]. Therefore, identifying predictors of adverse postoperative outcomes is imperative to improve (i) treatment decisions, (ii) perioperative management, and potentially, (iii) surgical outcomes among patients with CRC.

The skeletal muscle is a locomotory organ with secretory and metabolic properties [5] that has gained attention as a prognosticator of adverse outcomes in oncology [6]. An increasing body of evidence has shed light on the predictive value of the skeletal muscle in the surgical oncology setting [7, 8, 9, 10, 11, 12, 13]. Several studies have used abdominal computed tomography (CT) scans to opportunistically assess low skeletal muscle mass (SMM) and density (SMD) that are indicative of sarcopenia and myosteatosis (infiltration of fat in the skeletal muscle), respectively. Although low SMM or low SMD, most commonly at the third lumbar vertebra (L3), is often considered predictive of adverse postoperative outcomes among patients following CRC surgery, the findings are inconsistent. For example, some studies have demonstrated that CT‐based low skeletal muscle index (SMI), a measure of muscle quantity, was associated with severe postoperative complications [8, 12], higher LOS [8, 12, 13], and overall mortality (OM) following CRC resection than normal SMI [8, 11, 13, 14]. However, others found that CT‐derived low SMI was not a predictor of severe postoperative complications [9, 13, 14] nor was it a predictor of LOS [9, 14] or OM [9, 12] following CRC surgery. Regarding SMD, several studies have demonstrated a higher risk of severe postoperative complications [9, 12, 13], LOS [9, 12, 13, 14], and OM [9, 11, 12, 13], among patients with low SMD undergoing CRC surgery. Although low SMD appears to be a more consistent predictor of adverse postoperative outcomes following CRC surgery than low SMI, further research is warranted to confirm these findings. Additionally, most studies have examined SMI and SMD as binary variables [9, 11, 12, 13], using different cutoffs. This dichotomisation of participants into high/normal versus low SMI or SMD is of clinical relevance, but it precludes assessing whether and how changes in SMI or SMD affect the risk of the respective outcome of interest.

Elucidating the performance of SMI and SMD in predicting postoperative outcomes following CRC surgery may assist clinicians identifying high‐risk patients and inform preoperative strategies (e.g., prehabilitation) and monitoring to improve patient care and surgical outcomes. The primary aim of this study was to determine the associations of CT‐derived SMI and SMD with postoperative outcomes including LOS, occurrence of severe postoperative complications, discharge location, 90‐day mortality, and OM. The secondary aim was to examine the associations of each low SMI and low SMD based on two common definitions [13, 15] on the same postoperative outcomes following CRC surgery. We hypothesised that lower SMI and SMD, whether treated as continuous or binary (low vs. normal) variables, would each be independently associated with worse short‐ and long‐term postoperative outcomes.

## 2 Methods

### 2.1 Study Setting and Population

We conducted a retrospective cohort study of patients who underwent CRC surgery at the Our Lady of the Lake (OLOL) Hospital in Baton Rouge, Louisiana. Potentially eligible participants were identified through query of the OLOL tumor registry that included patients who were treated surgically for CRC at this institution from October 2018 until February 2024.

Participants were included if they had undergone surgery for CRC and had a preoperative abdominal CT scan at least 6 months prior to surgery. Exclusion criteria included (i) transanal and endoscopic resection of malignant polyps, (ii) CT artifacts, (iii) no evidence of malignancy and (iv) stage IV disease. Study data were retrieved from (i) the OLOL tumour registry, (ii) electronic medical records (EMR) and (iii) the Vizient Clinical Data Base (CDB). The Vizient CDB is a healthcare platform that gathers data from multiple hospitals nationwide to inform data‐driven decisions aiming to reduce healthcare costs and optimise patient outcomes and care. The OLOL tumour registry provided patients' EMR number and date of surgery for each patient. EMRs along with the Vizient CDB were used to retrieve patients' demographic and clinical characteristics, Charlson Comorbidity Index (CCI) [16], type of surgery, anthropometric characteristics before surgery, postoperative LOS, severe postoperative complications and discharge location. All study procedures were approved by the Institutional Research Board at Louisiana State University Health Sciences Center New Orleans (ID: 7224). The requirement for obtaining informed consent from study participants was waived due to the retrospective nature of the study. Data from the Vizient CDB were used with the permission of Vizient (all rights reserved).

### 2.2 Assessment of Skeletal Muscle Index and Skeletal Muscle Density

**FIGURE 1:**
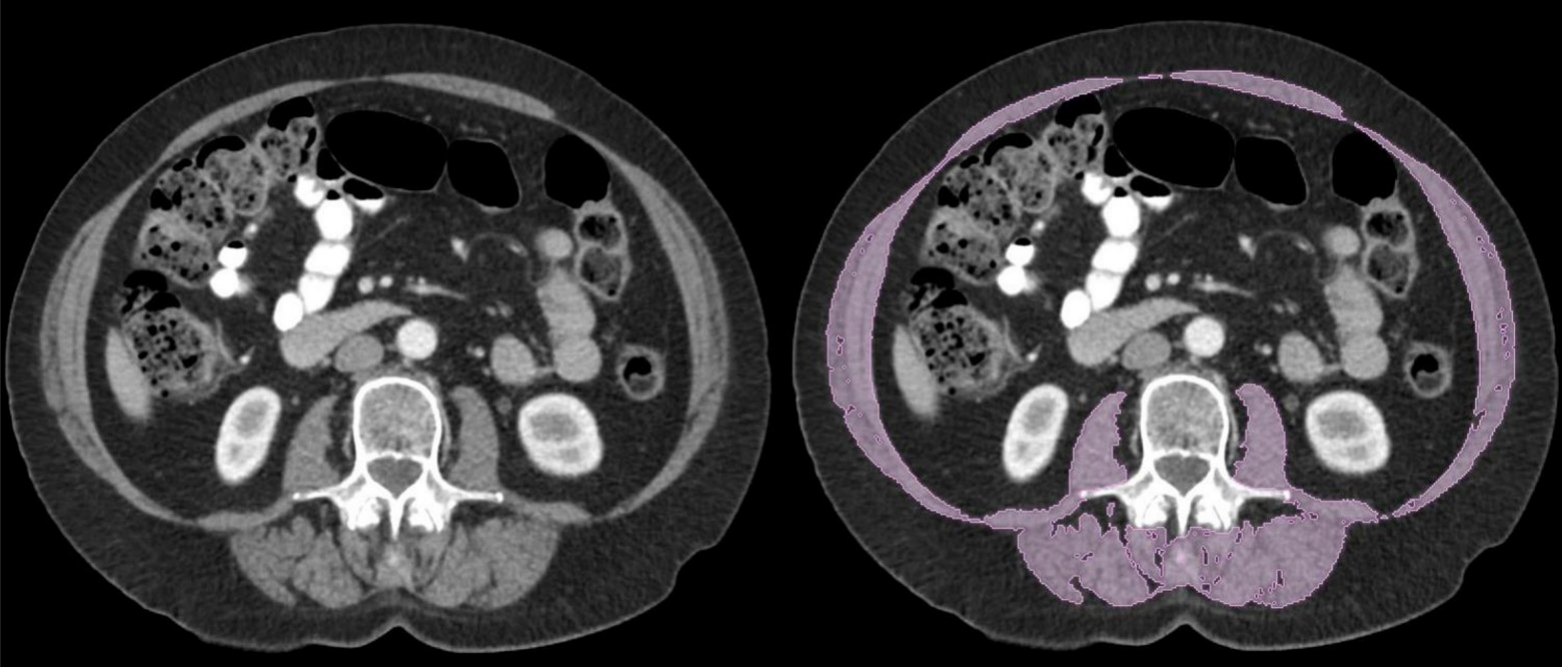
Skeletal muscle segmentation at the L3 level using Visage 7.1.18 (Visage Imaging Inc., San Diego, California, United States). The image on the left illustrates unsegmented skeletal muscle. The image on the right illustrates segmented skeletal muscle.

SMI and SMD were assessed at the third lumbar vertebra (L3) using a single abdominal CT scan prior to surgery. To quantify SMI and SMD, a semi‐automated HU threshold‐based segmentation approach was employed using our picture archiving and communication systems (PACS)‐integrated medical imaging software (Visage 7.1.18, Visage Imaging Inc., San Diego, California, United States). Muscle segmentation was performed by a member of the team with experience in radiographic tissue segmentation (EP). In line with previous work, the Hounsfield unit (HU) range to for the skeletal muscle was set between −29 and +150. [17] SMI was calculated by dividing the cross‐sectional area (CSA) (cm^2^) of the skeletal muscle at the L3 level by the patient's square of body height in meters (cm^2^/m^2^). SMD was expressed in HUs representing the average radiation attenuation of the skeletal muscle at the L3 level. The values for both the CSA and the average HU of the skeletal muscle were produced by Visage 7.1.18 following tissue segmentation (Figure 1).

Low SMI and low SMD were defined using two different definitions, the first by Martin et al. [15] and the second by Xiao et al. [13], which have been widely used in the CRC literature [7, 10, 12, 13, 18, 19, 20]. Per Martin et al. [15], the thresholds for low SMI for men and women with BMI < 25 kg/m^2^ were < 43 cm^2^/m^2^ and < 41 cm^2^/m^2^, respectively, whereas an SMI < 53 cm^2^/m^2^ was used for men with BMI ≥ 25 kg/m^2^. For women with BMI ≥25 kg/m^2^, the threshold for low SMI is also < 41 cm^2^/m^2^ [15]. Additionally, the threshold for low SMD was < 41 HU for patients with BMI < 25 kg/m^2^ and < 33 HU for those with BMI ≥ 25 kg/m^2^ [15]. Per Xiao et al. [13], the thresholds for low SMI in men and women with BMI < 30 were 52.3 cm^2^/m^2^ and < 38.6 cm^2^/m^2^, respectively. For men and women with BMI ≥30 kg/m^2^, the criteria for low SMI were < 54.3 cm^2^/m^2^ and < 46.6 cm^2^/m^2^, respectively. Using the same definition, the cutoffs for low SMD in men and women were < 35.5 HU and < 32.5 HU, respectively [13].

### 2.3 Outcome Measures

Study outcomes included (i) postoperative LOS, (ii) occurrence of severe postoperative complications up to 30 days after surgery, (iii) discharge location, (iv) 90‐day mortality and (v) OM. Postoperative LOS was measured in days from the day of surgery until the day of hospital discharge. Severe postoperative complications were defined as grade ≥ 3 complications using the Clavien–Dindo classification of surgical complications system [21]. Discharge location was dichotomised to (a) discharge to home, which was the reference category for this outcome, and (b) discharge to a non‐home location including skilled nursing facility, inpatient rehabilitation or long‐term care. Survival status was assessed up to 90 days from the day of surgery to determine 90‐day mortality. OM was also assessed from the day of surgery until death from any cause or loss to follow‐up before the end of data collection (April 30, 2024). Survival status during the study was confirmed through EMRs and obituary searches.

### 2.4 Statistical Analysis

The characteristics of study participants were summarised using the mean and standard deviation for continuous variables that were normally distributed, and the median and interquartile range for non‐normally distributed continuous variables. Frequencies and proportions were used for categorical data. Differences in age, sex, BMI, race and comorbidity burden were compared between patients included in the analysis and those excluded using an independent samples *t*‐test and chi‐squared tests, as appropriate. The intraclass correlation coefficient (ICC_3,1_) based on a two‐way mixed‐effects with absolute agreement was used to assess the repeatability of CT‐based measurements in a random subset of 62 patients (15% of the cohort). To examine the primary objective, SMI and SMD were used as continuous predictors within the same multivariable models and were expressed per 5‐unit decrease to enhance interpretability of the effect in line with previous work [22]. Quadratic terms were used to screen for potential non‐linearity in order to preserve model parsimony and maintain an acceptable events‐per‐variable ratio given the limited number of events in the study outcomes instead of other approaches (e.g., spline models) that require additional degrees of freedom.

To address the secondary objective, participants were dichotomised into low versus normal SMI, as well as low versus normal SMD based on two different definitions mentioned above [13, 15]. LOS was assessed using negative binomial regression. The occurrence of severe postoperative complications as well as discharge location were assessed using logistic regression. The time to 90‐day mortality and OM was examined using Cox regression. The probability of OM based on low SMI and low SMD was assessed separately using Kaplan–Meier plots and log‐rank tests, while the proportional hazard assumption was examined using visual techniques (i.e., no mild‐curve crossing). Covariates in multivariable analyses included age, sex, comorbidity burden per the Charlson Comorbidity Index, AJCC stage, neoadjuvant treatment status and type of surgery. However, the multivariable model for discharge location was only adjusted for age and comorbidity burden due to the small number of events. For each study outcome, three multivariable models were created adjusting for the same covariates. The first multivariable model for each outcome included SMI and SMD as continuous predictors. The second multivariable model included low SMI and low SMD using the criteria per Martin et al. [15], while the third multivariable model for each outcome included low SMI and low SMD using the criteria per Xiao et al. [13]. Collinearity was assessed using the variance inflation factor (VIF). Parametric correlations were assessed between continuous SMI and SMD, while non‐parametric correlations were used between low SMI and low SMD using different thresholds per Martin et al. [15] and Xiao et al. [13]. All analyses were completed using IBM SPSS for Windows, Version 29.0. Armonk, New York, United States: IBM Corp.

### 2.5 Sensitivity Analyses

Two sensitivity analyses were conducted to assess the robustness of the results from the main analysis. The first sensitivity analysis examined the associations between CT‐derived muscle characteristics and severe postoperative complications in multivariable models with fewer covariates (age, comorbidity burden, and type of surgery) to improve the events‐per‐variable ratio and preserve model parsimony.

The second sensitivity analysis was conducted to examine the associations between CT‐based skeletal muscle characteristics and study outcomes in patients with an available CT scan ≤ 2 months before surgery. This approach to minimize the time between CT and surgery improves classification of SMI and SMD status most proximal to surgery as disease‐related processes can promote muscle catabolism [6].

## 3 Results

A total of 881 patients had undergone CRC surgery from October 2018 to February 2024. Of these, 466 were excluded, leaving 415 participants for the analysis. The reasons for exclusion are described in the study participant flow chart (Supplemental Figure 1). No significant differences were found in age, BMI, sex, race and comorbidity burden between patients who were included in the analysis versus those excluded (Supplemental Table 1). The characteristics of the entire cohort are shown in Table 1. In brief, the mean age of participants was 63.7 years with a mean body mass index (BMI) of 28.2 kg/m^2^. Females comprised 45.5% of the cohort. Most participants were White (60.2%) while 36.4% were Black. The most common type of surgery was laparoscopic (39.8%) followed by robotic‐assisted (39.5%) and open (20.7%). The mean time from the preoperative CT scans to surgery was 16.2 days. The mean SMI and SMD in the entire cohort were 48.9 cm^2^/m^2^ and 34.6 HU, respectively. Using the criteria per Martin et al., 151 (36.4%) had low SMI while 210 (50.6%) had low SMD prior to surgery. Using the criteria per Xiao et al., 161 (38.8%) had low SMI while 202 (48.7%) had low SMD (Table 1 and Supplemental Table 2).

**TABLE 1:** Characteristics of study participants.

Characteristic	All participants (*n* = 415)
Age (years), mean (SD)	63.7 (13.2)
BMI (kg/m^2^), mean (SD)	28.2 (6.5)
Sex (females), *n* (%)	189 (45.5)
Race, *n* (%)	
White	250 (60.2)
Black	151 (36.4)
Other	14 (3.4)
Charlson Comorbidity Index, median (IQR)	2 (2–3)
Cancer site, *n* (%)	
Colon	314 (75.7)
Rectal	101 (24.3)
AJCC stage	
I	89 (21.4)
II	143 (34.5)
III	183 (44.1)
Neoadjuvant therapy	58 (14.0)
Type of surgery, *n* (%)	
Open	86 (20.7)
Laparoscopic	165 (39.8)
Robotic assisted	164 (39.5)
SMI (cm^2^/m^2^), mean (SD)	48.9 (11.4)
SMD (HU), mean (SD)	34.6 (10.2)
Cutoff criteria per Martin et al., *n* (%)	
Low SMI	151 (36.4)
Low SMD	210 (50.6)
Cutoff criteria per Xiao et al., *n* (%)	
Low SMI	161 (38.8)
Low SMD	202 (48.7)
Postoperative LOS (days), median (IQR)	4 (3–6)
Occurrence of grade ≥ 3 postoperative^a^ complications (yes), *n* (%)	50 (12.0)
Discharge location (non‐home), *n* (%)^b^	30 (7.2)
90‐day mortality (yes), *n* (%)	12 (2.9)
Overall mortality (yes), *n* (%)	75 (18.1)

Abbreviations: BMI, body mass index; HU, Hounsfield units; IQR, interquartile range; LOS, length of stay; SD, standard deviation; SMD, skeletal muscle density; SMI, skeletal muscle index.

^a^ Occurrence of grade ≥ 3 postoperative complications: *n* = 5 were not included due to missing data up to 30 days after surgery.

^b^ Discharge location (non‐home): *n* = 6 participants were removed from the analysis due to the following reasons: (i) *n* = 4 expired prior to discharge, (ii) *n* = 1 were discharged/transferred to court/law enforcement and (iii) *n* = 1 left against medical advice.

The ICC for the intra‐rater reliability in measuring SMI and SMD was 0.99 (95%CI 0.99–0.99) for both measures. No evidence of collinearity was observed for continuous or binary SMI and SMD in multivariable analyses based on VIF. The parametric correlation coefficient between continuous SMI and SMD was *r* = 0.43 (*p* < 0.001). The non‐parametric correlation between low SMI and low SMD per Martin et al. was *r* = 0.28 (*p* < 0.001). When using the criteria by Xiao et al., low SMI and low SMD exhibited a weak correlation (*r* = 0.26, *p* < 0.001). The quadratic terms for SMI and SMD as continuous predictors were not significant, suggesting no evidence of non‐linearity with study outcomes. The quadratic term for SMI per 5‐unit decrease reached statistical significance in the multivariable model of OM. Upon further examination, visual diagnostics did not indicate a meaningful deviation from a linear relationship. Therefore, SMI per 5‐unit decrease was retained in the multivariable model for OM for model parsimony.

### 3.1 Postoperative LOS

The median LOS among all participants was 4 days. The median LOS among participants stratified by SMI and SMD is shown in Supplemental Table 2. Table 2 lists the univariate and multivariable analyses on the associations between skeletal muscle characteristics and postoperative LOS. Each 5‐unit decrease in SMD was associated with a higher LOS in multivariable analyses (incidence rate ratio [IRR] 1.06, 95%CI 1.02–1.10, *p* = 0.003) (see multivariable model 1 in Table 2). After applying clinical cutoffs, patients with low SMD were expected to have a higher incidence of LOS compared to those with normal SMD using the criteria per Martin et al. (IRR 1.21, 95%CI 1.05–1.41, *p* = 0.010) or per Xiao et al. (IRR 1.28, 95%CI 1.11–1.48, *p* < 0.001) (see multivariable models 2 and 3 in Table 2). Although changes in SMI were predictive of higher LOS in the univariate analysis (IRR 1.05, 95%CI 1.02–1.08, *p* = 0.002), no significant associations were found in the multivariable analysis (IRR 1.02, 95%CI 0.98–1.05, *p* = 0.39). Similarly, low SMI was not significantly associated with LOS irrespective of the definition used in the adjusted analysis (Table 2).

**TABLE 2:** Associations between skeletal muscle characteristics and postoperative LOS (*n* = 415).

Variable	Univariate IRR (95%CI)	*p*	Multivariable IRR (95%CI)#1^a^	*p*	Multivariable IRR (95%CI)#2^b^	*p*	Multivariable IRR (95%CI)#3^c^	*p*
SMI (cm^2^/m^2^) per 5‐unit decrease	1.05 (1.02–1.08)	0.002	1.02 (0.98–1.05)	0.39	Not included		Not included	
SMD (HU) per 5‐unit decrease	1.09 (1.05–1.12)	<0.001	1.06 (1.02–1.10)	0.003	Not included		Not included	
Low SMI per Martin	1.05 (0.92–1.21)	0.47	Not included		0.91 (0.79–1.05)	0.20	Not included	
Low SMD per Martin	1.37 (1.20–1.56)	<0.001	Not included		1.21 (1.05–1.41)	0.010	Not included	
Low SMI per Xiao	1.14 (1.00–1.31)	0.049	Not included		Not included		1.01 (0.88–1.15)	0.91
Low SMD per Xiao	1.42 (1.25–1.62)	<0.001	Not included		Not included		1.28 (1.11–1.48)	<0.001

*Note:* Multivariable models are adjusted for age, sex, Charlson Comorbidity Index, tumour stage, neoadjuvant treatment status and type of surgery.

Abbreviations: HUs, Hounsfield units; SMD, skeletal muscle density; SMI, skeletal muscle index.

^a^ Multivariable model #1 includes SMI and SMD as continuous predictors, each per 5‐unit decrease.

^b^ Multivariable model #2 includes low SMI and low SMD (binary predictors) using the cutoffs per Martin et al.

^c^ Multivariable model #3 includes low SMI and low SMD (binary predictors) using the cutoffs per Xiao et al.

In the multivariable sensitivity analysis of patients with a CT scan ≤ 2 months before surgery, each 5‐unit decrease in SMD remained a significant predictor of higher LOS (IRR 1.05, 95%CI 1.01–1.09, *p* = 0.021) (Supplemental Table 3). Of the two definitions of low SMD, only the definition per Xiao et al. was significantly associated with higher LOS (IRR 1.21, 95%CI 1.04–1.40, *p* = 0.014), while the definition of low SMD per Martin et al. was attenuated and was no longer significant (IRR 1.13, 95%CI 0.97–1.31, *p* = 0.11) (Supplemental Table 3). In line with the main analysis, SMI as a continuous or binary predictor was not significantly associated with LOS in the multivariable sensitivity analysis.

### 3.2 Occurrence of Severe Postoperative Complications

A total of 50 (12.0%) participants experienced at least one severe postoperative complication defined as a grade ≥ 3 event per the Clavien–Dindo classification of surgical complications system. Decreases in each SMI and SMD were associated with greater odds of severe postoperative complications in univariate analysis (Table 3). In multivariable analysis, each 5‐unit decrease in SMD (OR 1.25, 95%CI 1.02–1.53, *p* = 0.029) but not in SMI (OR 1.09, 95%CI 0.90–1.32, *p* = 0.35) was significantly associated with the occurrence of severe postoperative complications (see multivariable model 1 in Table 3). Low SMI or low SMD per the definition by Martin et al. or Xiao et al. were not significantly associated with severe postoperative complications (see multivariable models 2 and 3 in Table 3).

**TABLE 3:** Associations between skeletal muscle characteristics and severe (grade ≥ 3) postoperative complications (*n* = 410).

Variable	Univariate OR (95%CI)	*p*	Multivariable OR (95%CI)#1^a^	*p*	Multivariable OR (95%CI)#2^b^	*p*	Multivariable OR (95%CI)#3^c^	*p*
SMI (cm^2^/m^2^) per 5‐unit decrease	1.22 (1.05–1.42)	0.009	1.09 (0.90–1.32)	0.35	Not included		Not included	
SMD (HU) per 5‐unit decrease	1.35 (1.16–1.57)	<0.001	1.25 (1.02–1.53)	0.029	Not included		Not included	
Low SMI per Martin	1.46 (0.80–2.66)	0.22	Not included		1.01 (0.51–2.00)	0.97	Not included	
Low SMD per Martin	2.61 (1.38–4.94)	0.003	Not included		1.67 (0.78–3.55)	0.19	Not included	
Low SMI per Xiao	1.29 (0.71–2.35)	0.39	Not included		Not included		0.95 (0.48–1.89)	0.89
Low SMD per Xiao	2.32 (1.25–4.32)	0.008	Not included		Not included		1.56 (0.74–3.30)	0.24

*Note:* Multivariable models are adjusted for age, sex, Charlson Comorbidity Index, tumour stage, neoadjuvant treatment status and type of surgery. Grade ≥ 3 postoperative complications were not assessed for five patients due to missing data up to 30 days after surgery.

Abbreviations: HUs, Hounsfield units; SMD, skeletal muscle density; SMI, skeletal muscle index.

^a^ Multivariable model #1 includes SMI and SMD as continuous predictors, each per 5‐unit decrease.

^b^ Multivariable model #2 includes low SMI and low SMD (binary predictors) using the cutoffs per Martin et al.

^c^ Multivariable model #3 includes low SMI and low SMD (binary predictors) using the cutoffs per Xiao et al.

Given the limited number of events and to preserve model parsimony, a sensitivity analysis with fewer covariates was conducted (Supplemental Table 4). SMD per 5‐unit decrease was significantly associated with severe postoperative complications (OR 1.23, 95%CI 1.02–1.49, *p* = 0.034), adjusting for age, comorbidity burden and type of surgery. No significant associations were found between low SMD and severe postoperative complications. Similarly, SMI as a continuous or binary predictor was not significantly associated with severe postoperative complications (Supplemental Table 4).

Another sensitivity analysis was conducted to include patients with an available CT scan ≤ 2 months before surgery (Supplemental Table 5). In line with the main analysis, each 5‐unit decrease in SMD was associated with higher odds of severe postoperative complications (OR 1.26, 95%CI 1.02–1.55, *p* = 0.030) in the adjusted analysis. However, low SMD was not significantly associated with severe postoperative complications regardless of the definition used. SMI as a continuous or binary variable did not predict severe postoperative complications (Supplemental Table 5).

### 3.3 Discharge Location

Of the 409 participants with available data on discharge location, *n* = 30 were discharged to a non‐home location including a skilled nursing facility, inpatient rehabilitation, or long‐term care, while *n* = 379 were discharged to home. A total of 6 participants were excluded from the analysis due to the following reasons: (i) *n* = 4 patients expired before discharge, (ii) *n* = 1 patient was discharged/transferred to court/law enforcement and (iii) *n* = 1 left against medical advice (Table 1). Table 4 lists the associations between skeletal muscle characteristics and non‐home discharge. Each 5‐unit decrease in SMI (OR 1.32, 95%CI 1.08–1.62, *p* = 0.007) or SMD (OR 1.63, 95%CI 1.34–1.99, *p* < 0.001) was predictive of discharge to a non‐home location after surgery in univariate analysis. However, in multivariable analysis, the significant associations with discharge to a non‐home location persisted only for every 5‐unit decrease in SMD (OR 1.40, 95%CI 1.09–1.79, *p* = 0.007) (see multivariable model 1 in Table 4). Additionally, low SMD using the definition by Martin et al. (OR 3.58, 95%CI 1.13–11.37, *p* = 0.030) or Xiao et al. (OR 8.55, 95%CI 1.01–11.49, *p* = 0.034) was significantly associated with non‐home discharge (see multivariable models 2 and 3 in Table 4). SMI was not prognostic of discharge location when treated as a continuous or binary predictor in adjusted analysis (Table 4).

**TABLE 4:** Associations between skeletal muscle characteristics and non‐home discharge (*n* = 409).

Variable	Univariate OR (95%CI)	*p*	Multivariable OR (95%CI)#1^a^	*p*	Multivariable OR (95%CI)#2^b^	*p*	Multivariable OR (95%CI)#3^c^	*p*
SMI (cm^2^/m^2^) per 5‐unit decrease	1.32 (1.08–1.62)	0.007	1.06 (0.85–1.32)	0.59	Not included		Not included	
SMD (HU) per 5‐unit decrease	1.63 (1.34–1.99)	<0.001	1.40 (1.09–1.79)	0.007	Not included		Not included	
Low SMI per Martin	2.48 (1.17–5.25)	0.018	Not included		1.29 (0.55–3.04)	0.97	Not included	
Low SMD per Martin	7.11 (2.44–20.77)	<0.001	Not included		3.58 (1.13–11.37)	0.030	Not included	
Low SMI per Xiao	2.56 (1.19–5.47)	0.015	Not included		Not included		1.25 (0.54–2.92)	0.60
Low SMD per Xiao	7.74 (2.65–22.61)	<0.001	Not included		Not included		8.55 (1.01–11.49)	0.034

*Note:* Multivariable models are adjusted for age and Charlson Comorbidity Index due to the small number of events (*n* = 30). Discharge location (non‐home): *n* = 6 participants were removed from the analysis due to the following reasons: (i) *n* = 4 expired prior to discharge, (ii) *n* = 1 was discharged/transferred to court/law enforcement and (iii) *n* = 1 left against medical advice.

Abbreviations: HUs, Hounsfield units; SMD, skeletal muscle density; SMI, skeletal muscle index.

^a^ Multivariable model #1 includes SMI and SMD as continuous predictors, each per 5‐unit decrease.

^b^ Multivariable model #2 includes low SMI and low SMD (binary predictors) using the cutoffs per Martin et al.

^c^ Multivariable model #3 includes low SMI and low SMD (binary predictors) using the cutoffs per Xiao et al.

In the multivariable sensitivity analysis of patients with a CT scan ≤ 2 months before surgery each 5‐unit decrease in SMD was significantly associated with non‐home discharge (OR 1.38, 95%CI 1.08–1.78, *p* = 0.011) (Supplemental Table 6). Nonetheless, the associations between low SMD and non‐home discharge were no longer significant (Supplemental Table 6). Similarly, SMI was not significantly associated with non‐home discharge in the multivariable analysis (Supplemental Table 6).

### 3.4 Ninety‐Day Mortality

A total of 12 (2.9%) of participants died during the first 90 days after surgery (Table 5). Given the small number of events, multivariable analysis was not feasible. In the univariate analysis, each 5‐unit decrease in SMD (HR 1.36, 95%CI 1.04–1.78, *p* = 0.024) was prognostic of 90‐day mortality (Table 5). No significant associations were found between SMI either as a continuous or binary predictor and 90‐day mortality. Similarly, low SMD was not prognostic of 90‐day mortality (Table 5).

**TABLE 5:** Univariate associations between skeletal muscle characteristics and 90‐day mortality (*n* = 415).

Variable	Univariate HR (95%CI)	*p*
SMI (cm^2^/m^2^) per 5‐unit decrease	1.32 (0.97–1.79)	0.074
SMD (HU) per 5‐unit decrease	1.36 (1.04–1.78)	0.024
Low SMI per Martin	2.53 (0.80–7.98)	0.11
Low SMD per Martin	3.01 (0.82–11.13)	0.098
Low SMI per Xiao	2.28 (0.72–7.18)	0.16
Low SMD per Xiao	3.25 (0.88–11.99)	0.077

Abbreviations: HUs, Hounsfield units; SMD, skeletal muscle density; SMI, skeletal muscle index.

These results persisted in the sensitivity analysis of patients with an available CT scan ≤ 2 months before surgery, where each 5‐unit decrease in SMD was significantly associated with a higher risk of 90‐day mortality (HR 1.34, 95%CI 1.03–1.75, *p* = 0.032) (Supplemental Table 7).

### 3.5 Overall Mortality

Over a median follow‐up of 31.6 months, 75 (18.1%) participants died. Table 6 lists the associations between skeletal muscle characteristics and OM. Each 5‐unit decrease in SMI was predictive of OM in univariate analysis (HR 1.18, 95%CI 1.05–1.31, *p* = 0.004), but this effect was blunted in the multivariable analysis (HR 1.05, 95%CI 0.92–1.20, *p* = 0.49). Each 5 unit‐decrease in SMD was associated with a 26% higher risk of OM (HR 1.26, 95%CI 1.09–1.46, *p* = 0.002) in the adjusted analysis (see multivariable model 1 in Table 6). Subsequently, SMI and SMD were treated as binary variables using two different cutoffs for each [13, 15]. Using the criteria per Martin et al., 40 patients with low SMI died of any cause, compared to 35 deaths in the normal SMI group (Supplemental Table 2). Similarly, of the 75 deaths, 55 occurred among patients with low SMD and 20 among those with normal SMD per the definition by Martin et al. (Supplemental Table 2). Using the criteria per Xiao et al., 42 patients with low SMI died relative to 33 deaths in the normal SMI group, while a total of 55 deaths occurred in the low SMD group compared to 20 deaths in patients with normal SMD (Supplemental Table 2).

**TABLE 6:** Associations between skeletal muscle characteristics and overall mortality (*n* = 415).

Variable	Univariate HR (95%CI)	*p*	Multivariable HR (95%CI)#1^a^	*p*	Multivariable HR (95%CI)#2^b^	*p*	Multivariable HR (95%CI)#3^c^	*p*
SMI (cm^2^/m^2^) per 5‐unit decrease	1.18 (1.05–1.31)	0.004	1.05 (0.92–1.20)	0.49	Not included		Not included	
SMD (HU) per 5‐unit decrease	1.41 (1.26–1.57)	<0.001	1.26 (1.09–1.46)	0.002	Not included		Not included	
Low SMI per Martin	2.33 (1.48–3.67)	<0.001	Not included		1.65 (0.98–2.75)	0.057	Not included	
Low SMD per Martin	3.10 (1.86–5.18)	<0.001	Not included		1.93 (1.08–3.43)	0.027	Not included	
Low SMI per Xiao	2.21 (1.35–3.35)	0.001	Not included		Not included		1.36 (0.83–2.25)	0.23
Low SMD per Xiao	3.36 (2.01–5.61)	<0.001	Not included		Not included		1.72 (0.96–3.10)	0.070

*Note:* Multivariable models are adjusted for age, sex, Charlson Comorbidity Index, tumour stage, neoadjuvant treatment status and type of surgery.

Abbreviations: HUs, Hounsfield units; SMD, skeletal muscle density; SMI, skeletal muscle index.

^a^ Multivariable model #1 includes SMI and SMD as continuous predictors, each per 5‐unit decrease.

^b^ Multivariable model #2 includes low SMI and low SMD (binary predictors) using the cutoffs per Martin et al.

^c^ Multivariable model #3 includes low SMI and low SMD (binary predictors) using the cutoffs per Xiao et al.

**FIGURE 2:**
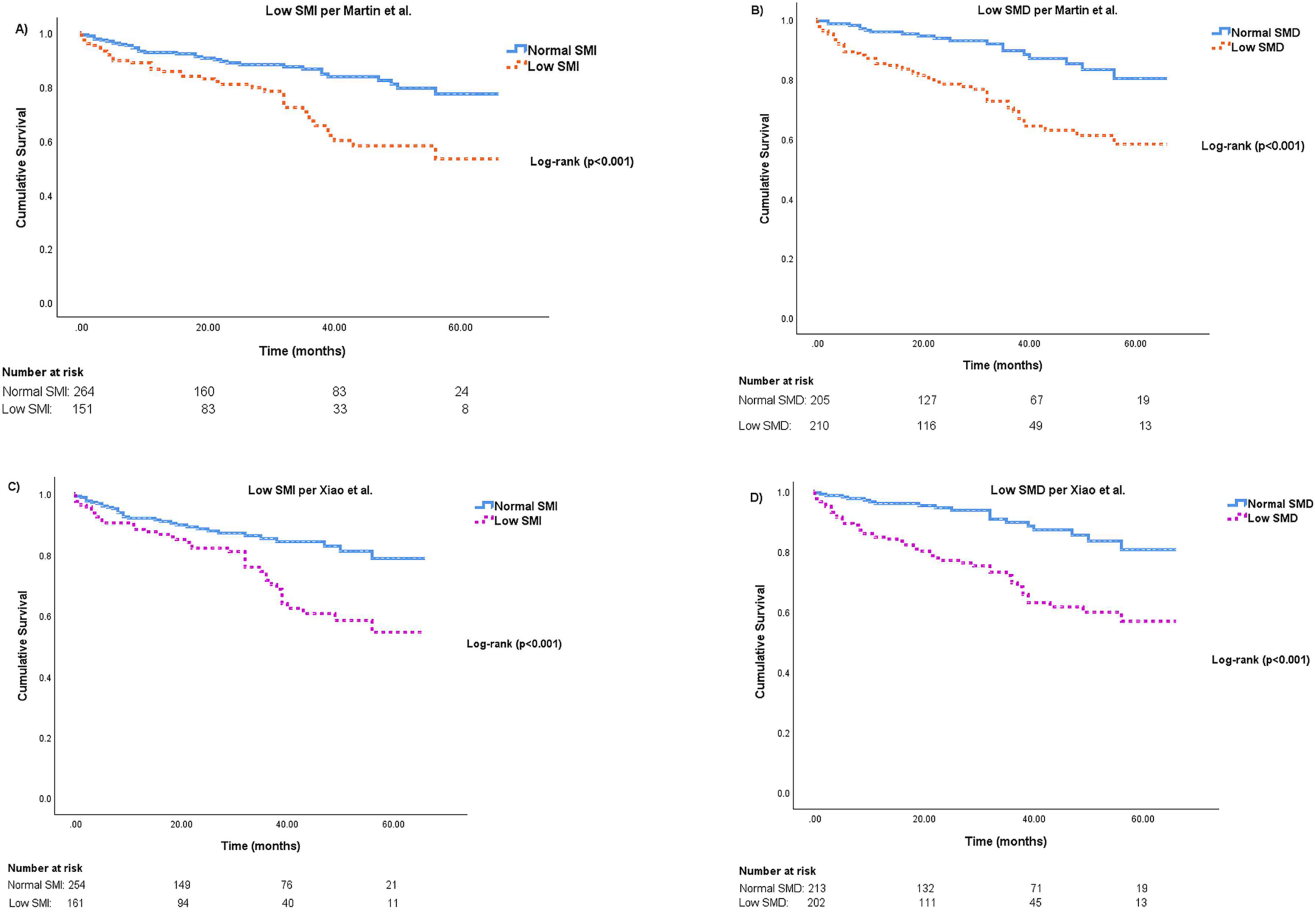
Probability of all‐cause mortality based on (A) low skeletal muscle index (SMI) and (B) low skeletal muscle density (SMD) per Martin et al., and (C) low SMI and (D) low SMD per Xiao et al. Using the criteria per Martin et al., the univariate hazard ratio (HR) for low SMI compared with normal SMI was HR = 2.33, 95%CI 1.48–3.67, *p* < 0.001, while the univariate HR for low SMD compared with normal SMD was HR = 3.10, 95%CI 1.86–5.18, *p* < 0.001. Using the criteria per Xiao et al., the univariate HR for low SMI compared with normal SMI was HR = 2.21, 95%CI 1.35–3.35, *p* < 0.001, while the univariate HR for low SMD compared with normal SMD was HR = 3.36, 95%CI 2.01–5.61, *p* < 0.001).

Figure 2A,B illustrates the probability of OM by SMI and SMD status using the criteria per Martin et al. According to the log‐rank test, the probability of OM was significantly higher in patients with low SMI than in those with normal SMI (*p* < 0.001). Similarly, the probability of OM was significantly higher in patients with low SMD compared to patients with normal SMD (log‐rank test *p* < 0.001) (Figure 2B). Figure 2C,D illustrates the probability of OM by SMI and SMD status using the criteria per Xiao et al. The probability of OM was higher in patients with low SMI (log‐rank test *p* < 0.001) and those with low SMD (log‐rank test *p* < 0.001).

In adjusted Cox regression, low SMD was significantly associated with OM only based on the criteria per Martin et al. (HR 1.93, 95%CI 1.08–3.43, *p* = 0.027) (see multivariable model 2 in Table 6). Low SMI was not significantly associated with OM (Table 6).

In the adjusted sensitivity analysis of patients with a CT scan ≤ 2 months before surgery, each 5‐unit decrease in SMD was significantly associated with OM (HR 1.27, 95%CI 1.09–1.48, *p* = 0.001) (Supplemental Table 8). Similarly low SMD using the criteria per Martin et al. remained a significant predictor of OM (HR 1.84, 95%CI 1.01–3.36, *p* = 0.046). Although the association between low SMD using the criteria per Xiao and OM was strengthened in the sensitivity analysis, it did not reach statistical significance (HR 1.80, 95%CI 0.98–3.30, *p* = 0.058). Low SMI was not significantly associated with OM in the sensitivity analysis regardless of the definition used (Supplemental Table 8).

## 4 Discussion

This study aimed to investigate the associations of CT‐derived SMI and SMD with postoperative outcomes following CRC surgery. Additionally, the study examined the associations of each low SMI and low SMD with postoperative outcomes using two different cutoffs [13, 15] per prior studies [7, 10, 12, 13, 18, 19]. SMD, either as a continuous or binary variable, predicted LOS and OM. Additionally, each 5‐unit decrease in SMD was significantly associated with severe postoperative complications, non‐home discharge, and 90‐day mortality. On the contrary, SMI was not predictive of short‐ or long‐term postoperative outcomes.

In our study, a 5‐unit decrease in SMD was significantly associated with a higher postoperative LOS. These findings are in line with those by Kemper et al. who found that SMD per 10‐unit increase was significantly associated with shorter postoperative LOS among patients following CRC surgery [9]. Notably, SMI as a continuous variable was not a significant predictor of LOS in our study nor in the study by Kemper et al. [9] After applying clinical cutoffs using two different definitions [13, 15], SMD remained an independent predictor of LOS, in line with previous work among patients undergoing CRC resection [12, 13]. However, in the sensitivity analysis that included patients with an available CT scan ≤ 2 months before surgery, only the definition of low SMD per Xiao et al. remained a significant predictor of LOS. Low SMI was not significantly associated with LOS in our cohort, contrary to previous studies that demonstrated an increased LOS in participants with low SMI after CRC surgery [8, 12, 13].

In the present cohort, each 5‐unit decrease in SMD was significantly associated with 25% greater odds of severe postoperative complications, results that persisted in sensitivity analyses. Our findings are in line with previous research, which demonstrated a negative relationship between SMD and severity of postoperative complications following CRC resection [9]. However, SMD as a binary variable was not significantly associated with severe postoperative complications in our cohort regardless of the definition used. However, previous work by Xiao et al. has demonstrated the prognostic value of SMD for severe postoperative complications. In a study of 1630 patients undergoing colon cancer surgery, those with low SMD were 2.4 times more likely to experience severe postoperative complications than those with normal SMD [13]. However, the study by Xiao et al. included only patients with colon cancer undergoing endoscopic polypectomy or laparoscopy while their adjusted analysis did not include surgical approach as a covariate, which could, in part, explain the divergent findings relative to our study despite using the same cutoffs. Additionally, low SMI in our study was not a significant predictor of severe postoperative complications. However, findings on the associations between low SMI and severe postoperative complications following CRC are inconsistent [7, 8, 12, 13].

Evidence on the associations between skeletal muscle characteristics and discharge location after CRC surgery is scarce. In a study of 816 patients with CRC, low SMI and low SMD were each associated with discharge to a location other than home [12]. In our study, each 5‐unit decrease in SMD was significantly associated with 40% greater odds of non‐home discharge, results that persisted in the sensitivity analysis. Although low SMD was significantly associated with non‐home discharge in the primary analysis, it was no longer significant in the sensitivity analysis, which may be attributed, in part, to the limited number of events and the reduction of sample size. Non‐home discharge may worsen patient quality of life and increase health care costs among different surgical populations [23, 24].

SMD as a continuous variable was also significantly associated with a higher risk of 90‐day mortality. Specifically, each 5‐unit decrease in SMD was significantly associated with a higher risk of death during the first 90 days after surgery, an effect that persisted in the sensitivity analysis. However, caution is advised when interpreting these findings due to the small number of events (*n* = 12) that did not enable us to perform multivariable analysis. Previous work has demonstrated that low SMI as well as the combination of low SMI and low SMD predicted short‐term mortality after colon cancer surgery [13].

Regarding OM, our results show that for every 5‐unit decrease in SMD the risk of death from any cause increases by 26%. In line with our results, Kemper et al. found that each 10 HU increase in SMD was associated with improved overall survival (HR 0.63, 95%CI 0.49‐0.81) in patients with CRC [9]. Low SMD using the criteria per Martin et al. was associated with a 93% higher risk of OM compared to normal SMD, in line with previous work [11, 13]. In the sensitivity analysis of patients with an available CT scan ≤ 2 months before surgery, the association between low SMD per Martin et al. and OM was attenuated but borderline significance (*p* = 0.046) was reached. Using the criteria per Xiao et., low SMD was not significantly associated with OM. Additionally, SMI was not predictive of OM in the adjusted analysis.

Our findings suggest that myosteatosis measured via CT‐based SMD appears to be a stronger predictor of adverse postoperative outcomes than SMI. SMI is a measure of muscle quantity which decreases as a result of aging and other causes (e.g., disuse event, diseases) [6], while SMD is a measure of myosteatosis that describes the accumulation of fat in the muscle [25, 26]. Given the nature of this study, exploration of potential mechanisms was not feasible. However, as it has been suggested [26], myosteatosis worsens the age‐related effects on the skeletal muscle, thereby contributing to declines in muscle strength and function [26, 27]. Notably, myosteatosis based on SMD exhibits stronger associations with muscle strength, physical performance [28], frailty [29], and comorbidity burden [30] than SMI (i.e., muscle size) in older adults [28] and patients with cancer [29, 30]. Additionally, myosteatosis is associated with inflammation [14, 31] and insulin resistance [32, 33]. Collectively, these mechanisms may, in part, explain why low SMD may be a stronger predictor of adverse postoperative outcomes than low SMI in patients with cancer [9, 34].

Interventions for increasing SMD mainly include exercise training. An increase in SMD reflects a decrease in fat accumulation in the skeletal muscle. Meta‐analytic data demonstrate that aerobic and resistance training improve SMD in adults [35]. However, evidence among patients with cancer, particularly in the surgical oncology setting, is scarce. Studies to examine changes in SMD following prehabilitation in patients awaiting cancer surgery are warranted.

An important strength of this study is the use of SMI and SMD as continuous and binary predictors. Most studies have examined SMI and SMD as binary predictors. Although this approach has clinical relevance, it limits the understanding of how changes in SMI or SMD affect the respective outcome(s) of interest. An important limitation of this study is the relatively sample size and the limited number of events, particularly for severe postoperative complications and discharge location, underscoring the need for cautious interpretation. As a result of these limitations, we were unable to determine whether the associations between CT‐based muscle characteristics and postoperative outcomes differ by cancer site in multivariable analyses. Another limitation is the inclusion of patients with contrast‐enhanced and non‐contrast CT scans. Specifically, of the 415 patients, 96% had contrast‐enhanced CT in line with the standard of care for CRC. The use of contrast results in a modest overestimation of SMD which may underdiagnose myosteatosis. However, the cutoff values for SMD that were used per Martin et al. were defined on contrast‐enhanced and unenhanced CT scans of patients with mixed solid malignancies [15]. Regarding the second definition of low SMD per Xiao et al., whether low SMD was derived based on contrast‐enhanced and/or unenhanced CT scans is unclear, as the authors did not specify [13]. Given the limited number of events and to preserve model parsimony, quadratic terms were used to screen for potential non‐linearity. However, the possibility of more complex non‐linear relationships cannot be excluded, and results should be interpreted with caution regarding the assumption of linearity. Additional limitations include the lack of documented physical activity and nutritional data, as well as measures of frailty throughout the study period which could influence the predictor and outcome variables.

## 5 Conclusion

Our findings suggest that SMD is a stronger predictor of postoperative outcomes than SMI following CRC surgery. The use of preoperative SMD may assist clinicians with risk stratification to guide treatment decisions and perioperative patient care following CRC diagnosis. Future studies should assess whether prehabilitation can improve SMD prior to CRC surgery.

## Funding

The authors have nothing to report.

## Conflicts of Interest

MingDe Lin is an employee and stockholder at Visage Imaging Inc. The rest of the authors have no competing interests to declare that are relevant to the content of this article.

## Supporting information


**Figure S1:** Flow diagram of study participants.
**Table S1:** Comparison between included and excluded patients.
**Table S2:** Characteristics of study participants by SMI and SMD using two different cutoff criteria (n = 415).
**Table S3:** Sensitivity analysis of the associations between skeletal muscle characteristics and postoperative LOS in patients with an available CT scan ≤ 2 months before surgery (n = 381).
**Table S4:** Sensitivity analysis of the multivariable associations between skeletal muscle characteristics and severe (grade ≥ 3) postoperative complications adjusting for fewer covariates to improve model parsimony (n = 410).
**Table S5:** Sensitivity analysis of the associations between skeletal muscle characteristics and severe (grade ≥ 3) postoperative complications in patients with an available CT scan ≤ 2 months before surgery (n = 376).
**Table S6:** Sensitivity analysis of the associations between skeletal muscle characteristics and non‐home discharge in patients with an available CT scan ≤ 2 months before surgery (n = 375).
**Table S7:** Sensitivity analysis of the univariate associations between skeletal muscle characteristics and 90‐day mortality in patients with an available CT scan ≤ 2 months before surgery (n = 381).
**Table S8:** Sensitivity analysis of the associations between skeletal muscle characteristics and overall mortality in patients with an available CT scan ≤ 2 months before surgery (n = 381).
