# Supplementary material for: Associations of CT‐Derived Skeletal Muscle Index and Density With Postoperative Outcomes Following Colorectal Cancer Surgery
Source: J Cachexia Sarcopenia Muscle. 2026 Sep 23;17(5):e70384. doi: 10.1002/jcsm.70384 (PMC13601755; doi:10.1002/jcsm.70384)
Supplement: Supplementary file 1 [file JCSM-17-e70384-s001.docx]

**Associations of CT-derived skeletal muscle index and density with postoperative outcomes following colorectal cancer surgery**

Efthymios Papadopoulos, PhD^1^, Brian A. Irving, PhD^1^, Guillaume Spielmann, PhD^1^, MingDe Lin, PhD^2,3^, Kelly R. Finan, MD^4^

**Affiliations**

^1^School of Kinesiology, Louisiana State University, Baton Rouge, LA, USA

^2^Department of Radiology and Biomedical Imaging, Yale University, New Haven, CT, USA

^3^Visage Imaging, Inc. San Diego, California, US

^4^Our Lady of the Lake Medical Center, Baton Rouge, LA, USA

**Corresponding author:**

Efthymios Papadopoulos, PhD

Assistant Professor

School of Kinesiology

Louisiana State University

50 Field House Drive, Suite 2140

Baton Rouge, 70803 LA

Email: [epap@lsu.edu](mailto:epap@lsu.edu)

Phone: 225-578-0938

Supplemental Figures and Tables

Total number of participants in clinic lists (n=881)

Total number of participants included (n=415)

Excluded:

- n= 102 transanal or endoscopic procedures (TAMIS, polypectomy)
- n= 20 duplicates
- n= 37 CT artifacts
- n= 209 did not have an available preoperative CT performed at OLOL
- n= 55 had no stage I-III disease^a^
- n= 15 no surgical procedure or had surgery unrelated to CRC
- n= 28 had stage IV disease

**Supplemental Figure 1.** Flow diagram of study participants

CRC: colorectal cancer; CT: computed tomography; OLOL: Our Lady of the Lake Medical Center; TAMIS: transanal minimally invasive surgery

^a^Of the 55 patients without stage I-III disease, 35 had no residual malignancy after a biopsy, 7 had carcinoma in situ, while 13 exhibited a pathologic complete response to neoadjuvant chemotherapy (i.e., no evidence of residual disease).

**Supplemental Table 1. Comparison between included and excluded patients**

| **Characteristic** | **Included patients (n=415)** | **Excluded patients**  **(n=431)^a^** | ***p*** |
| --- | --- | --- | --- |
| Age (years), mean (SD) | 63.7 (13.2) | 63.7 (12.8) | 0.96 |
| BMI (kg/m^2^), mean (SD) | 28.2 (6.5) | 29.1 (7.8) | 0.074 |
| Sex (females), n (%) | 189 (45.5) | 188 (43.6) | 0.57 |
| Race, n (%) |  |  | 0.77 |
| White | 250 (60.2) | 259 (60.0) |  |
| Black | 151 (36.4) | 160 (37.1) |  |
| Other | 14 (3.4) | 12 (2.8) |  |
| Charlson Comorbidity Index, mean (SD) | 3.1 (1.7) | 3.3 (2.4) | 0.055 |

^a^Patients who did not have surgery or those who had a surgical procedure unrelated to colorectal cancer were not included in this comparison (n=15).

**Supplemental Table 2. Characteristics of study participants by SMI and SMD using two different cutoff criteria (n=415).**

|  | **Cutoff criteria per Martin et al.** | | | | **Cutoff criteria per Xiao et al.** | | | |
| --- | --- | --- | --- | --- | --- | --- | --- | --- |
|  | SMI | | SMD | | **SMI** | | **SMD** | |
| Characteristic | Low  (n=151) | Normal  (n=264) | Low  (n=210) | Normal  (n=205) | Low  (n=161) | Normal  (n=254) | Low  (n=202) | Normal  (n=213) |
| Age (years), mean (SD) | 70.1 (12.6) | 59.9 (12.0) | 69.2 (11.6) | 57.9 (12.2) | 69.1 (12.8) | 60.2 (12.2) | 70.2 (10.9) | 57.5 (12.0) |
| BMI (kg/m^2^), mean (SD) | 26.2 (5.5) | 29.3 (6.8) | 28.2 (7.1) | 28.2 (5.9) | 25.3 (5.2) | 30.1 (6.6) | 29.1 (6.8) | 27.3 (6.1) |
| Sex (females), n (%) | 81 (53.6) | 108 (40.9) | 130 (61.9) | 59 (28.8) | 62 (38.5) | 127 (50.0) | 114 (56.4) | 75 (35.2) |
| Race, n (%) |  |  |  |  |  |  |  |  |
| White | 102 (67.5) | 148 (56.1) | 149 (71.0) | 101 (49.3) | 107 (66.5) | 143 (56.3) | 144 (71.3) | 106 (49.8) |
| Black | 45 (29.8) | 106 (40.2) | 58 (27.6) | 93 (45.4) | 49 (30.4) | 102 (40.2) | 54 (26.7) | 97 (45.5) |
| Other | 4 (2.6) | 10 (3.8) | 3 (1.4) | 11 (5.4) | 5 (3.1) | 9 (3.5) | 4 (2.0) | 10 (4.7) |
| Charlson Comorbidity Index, mean (SD) | 3.1 (1.6) | 3.0 (1.7) | 3.3 (1.8) | 2.8 (1.4) | 3.2 (1.8) | 2.9 (1.5) | 3.4 (1.9) | 2.7 (1.3) |
| Cancer site, n (%) |  |  |  |  |  |  |  |  |
| Colon | 123 (81.5) | 191 (72.3) | 161 (76.7) | 153 (74.6) | 123 (76.4) | 191 (75.2) | 160 (79.2) | 154 (72.3) |
| Rectal | 28 (18.5) | 73 (27.7) | 49 (23.3) | 52 (25.4) | 38 (23.6) | 63 (24.8) | 42 (20.8) | 59 (27.7) |
| AJCC stage |  |  |  |  |  |  |  |  |
| I | 25 (16.6) | 64 (24.2) | 42 (20.0) | 47 (22.9) | 30 (18.6) | 59 (23.2) | 38 (18.8) | 51 (23.9) |
| II | 55 (36.4) | 88 (33.3) | 74 (35.2) | 69 (33.7) | 62 (38.5) | 81 (31.9) | 71 (35.1) | 72 (33.8) |
| III | 71 (47.0) | 112 (42.4) | 94 (44.8) | 89 (43.4) | 69 (42.9) | 114 (44.9) | 93 (46.0) | 90 (42.3) |
| Neoadjuvant therapy | 17 (11.3) | 41 (15.5) | 28 (13.3) | 30 (14.6) | 23 (14.3) | 35 (13.8) | 22 (10.9) | 36 (16.9) |
| Type of surgery, n (%) |  |  |  |  |  |  |  |  |
| Open | 38 (25.2) | 48 (18.2) | 51 (24.3) | 35 (17.1) | 37 (23.0) | 49 (19.3) | 49 (24.3) | 37 (17.4) |
| Laparoscopic | 65 (43.0) | 100 (37.9) | 84 (40.0) | 81 (39.5) | 70 (43.5) | 95 (37.4) | 83 (41.1) | 82 (38.5) |
| Robotic assisted | 48 (31.8) | 116 (43.9) | 75 (35.7) | 89 (43.4) | 54 (33.5) | 110 (43.3) | 70 (34.7) | 94 (44.1) |
| SMI (cm^2^/m^2^), mean (SD) | 39.6 (6.7) | 54.2 (10.1) | 44.1 (9.8) | 53.9 (10.8) | 40.8 (7.1) | 53.9 (10.7) | 45.1 (10.5) | 52.5 (11.1) |
| SMD (HU), mean (SD) | 29.6 (9.6) | 37.5 (9.5) | 26.7 (7.2) | 42.7 (5.4) | 31.3 (10.4) | 36.7 (9.6) | 26.0 (6.6) | 42.7 (5.2) |
| Postoperative LOS (days), median (IQR) | 4 (3-6) | 4 (3-5) | 4 (3-6) | 3 (3-5) | 4 (3-6) | 4 (3-5) | 4 (3-7) | 3 (2-5) |
| Occurrence of grade ≥3 postoperative^a^ complications (yes), n (%) | 22 (14.6) | 28 (10.6) | 35 (16.7) | 15 (7.3) | 22 (13.7) | 28 (11.0) | 33 (16.3) | 17 (8.0) |
| Discharge location (non-home), n (%)^b^ | 17 (11.3) | 13 (4.9) | 26 (12.4) | 4 (2.0) | 18 (11.2) | 12 (4.7) | 26 (12.9) | 4 (1.9) |
| 90-day mortality (yes), n (%) | 7 (4.6) | 5 (1.9) | 9 (4.3) | 3 (1.5) | 3 (1.9) | 9 (3.5) | 8 (4.0) | 4 (1.9) |
| Overall mortality (yes), n (%) | 40 (26.5) | 35 (13.3) | 55 (26.2) | 20 (9.8) | 42 (26.1) | 33 (13.0) | 55 (27.2) | 20 (9.4) |

BMI: body mass index; HU: Hounsfield units; IQR: interquartile range; LOS: length of stay; SD: standard deviation; SMD: skeletal muscle density; SMI: skeletal muscle index

^a^Occurrence of grade ≥3 postoperative complications: n=5 were not included due to missing data up to 30 days after surgery.

^b^Discharge location (non-home): n=6 participants were removed from the analysis due to the following reasons: i) n=4 expired prior to discharge, ii) n=1 were discharged/transferred to court/law enforcement, and iii) n=1 left against medical advice.

**Supplemental Table 3. Sensitivity analysis of the associations between skeletal muscle characteristics and postoperative LOS in patients with an available CT scan ≤2 months before surgery (n=381)**

| **Variable** | **Univariate IRR (95%CI)** | ***p*** | **Multivariable**  **IRR (95%CI)#1^a^** | ***p*** | **Multivariable**  **IRR (95%CI)#2^b^** | ***p*** | **Multivariable**  **IRR (95%CI)#3^c^** | ***p*** |
| --- | --- | --- | --- | --- | --- | --- | --- | --- |
| SMI (cm^2^/m^2^)  per 5-unit decrease | 1.05 (1.02-1.08) | 0.003 | 1.00 (0.97-1.04) | 0.82 | Not included |  | Not included |  |
| SMD (HU)  per 5-unit decrease | 1.08 (1.05-1.12) | <0.001 | 1.05 (1.01-1.09) | 0.021 | Not included |  | Not included |  |
| Low SMI per Martin | 1.08 (0.94-1.25) | 0.25 | Not included |  | 0.94 (0.81-1.08) | 0.36 | Not included |  |
| Low SMD per Martin | 1.32 (1.16-1.51) | <0.001 | Not included |  | 1.13 (0.97-1.31) | 0.11 | Not included |  |
| Low SMI per Xiao | 1.07 (0.94-1.23) | 0.31 | Not included |  | Not included |  | 0.95 (0.83-1.09) | 0.47 |
| Low SMD per Xiao | 1.36 (1.19-1.56) | <0.001 | Not included |  | Not included |  | 1.21 (1.04-1.40) | 0.014 |

HUs: Hounsfield units; SMD: skeletal muscle density; SMI: skeletal muscle index

Note: Multivariable models are adjusted for age, sex, Charlson Comorbidity Index, tumor stage, neoadjuvant treatment status, and type of surgery

^a^Multivariable model #1 includes SMI and SMD as continuous predictors, each per 5-unit decrease

^b^Multivariable model #2 includes low SMI and low SMD (binary predictors) using the cutoffs per Martin et al.

^c^Multivariable model #3 includes low SMI and low SMD (binary predictors) using the cutoffs per Xiao et al.

**Supplemental Table 4. Sensitivity analysis of the multivariable associations between skeletal muscle characteristics and severe (grade≥3) postoperative complications adjusting for fewer covariates to improve model parsimony (n=410)**

| **Variable** | **Multivariable**  **OR (95%CI)#1^a^** | ***p*** | **Multivariable**  **OR (95%CI)#2^b^** | ***p*** | **Multivariable**  **OR (95%CI)#3^c^** | ***p*** |
| --- | --- | --- | --- | --- | --- | --- |
| SMI (cm^2^/m^2^)  per 5-unit decrease | 1.09 (0.93-1.29) | 0.26 | Not included |  | Not included |  |
| SMD (HU)  per 5-unit decrease | 1.23 (1.02-1.49) | 0.034 | Not included |  | Not included |  |
| Low SMI per Martin | Not included |  | 0.99 (0.50-1.96) | 0.98 | Not included |  |
| Low SMD per Martin | Not included |  | 1.91 (0.93-3.92) | 0.079 | Not included |  |
| Low SMI per Xiao | Not included |  | Not included |  | 0.88 (0.46-1.72) | 0.73 |
| Low SMD per Xiao | Not included |  | Not included |  | 1.64 (0.79-3.40) | 0.18 |

HUs: Hounsfield units; SMD: skeletal muscle density; SMI: skeletal muscle index

Note: Multivariable models were adjusted for age, Charlson Comorbidity Index, and type of surgery to improve parsimony and model estimates.

^a^Multivariable model #1 includes SMI and SMD as continuous predictors, each per 5-unit decrease

^b^Multivariable model #2 includes low SMI and low SMD (binary predictors) using the cutoffs per Martin et al.

^c^Multivariable model #3 includes low SMI and low SMD (binary predictors) using the cutoffs per Xiao et al.

**Supplemental Table 5. Sensitivity analysis of the associations between skeletal muscle characteristics and severe (grade≥3) postoperative complications in patients with an available CT scan ≤2 months before surgery (n=376)**

| **Variable** | **Univariate OR (95%CI)** | ***p*** | **Multivariable**  **OR (95%CI)#1^a^** | ***p*** | **Multivariable**  **OR (95%CI)#2^b^** | ***p*** | **Multivariable**  **OR (95%CI)#3^c^** | ***p*** |
| --- | --- | --- | --- | --- | --- | --- | --- | --- |
| SMI (cm^2^/m^2^)  per 5-unit decrease | 1.19 (1.03-1.34) | 0.021 | 1.05 (0.87-1.28) | 0.59 | Not included |  | Not included |  |
| SMD (HU)  per 5-unit decrease | 1.33 (1.14-1.55) | <0.001 | 1.26 (1.02-1.55) | 0.030 | Not included |  | Not included |  |
| Low SMI per Martin | 1.51 (0.81-2.81) | 0.19 | Not included |  | 1.14 (0.56-2.32) | 0.72 | Not included |  |
| Low SMD per Martin | 2.25 (1.17-4.32) | 0.015 | Not included |  | 1.37 (0.62-3.04) | 0.44 | Not included |  |
| Low SMI per Xiao | 1.05 (0.56-1.99) | 0.87 | Not included |  | Not included |  | 0.77 (0.37-1.59) | 0.48 |
| Low SMD per Xiao | 2.12 (1.11-4.03) | 0.022 | Not included |  | Not included |  | 1.54 (0.70-3.38) | 0.28 |

HUs: Hounsfield units; SMD: skeletal muscle density; SMI: skeletal muscle index

Note: Multivariable models are adjusted for age, sex, Charlson Comorbidity Index, tumor stage, neoadjuvant treatment status, and type of surgery

^a^Multivariable model #1 includes SMI and SMD as continuous predictors, each per 5-unit decrease

^b^Multivariable model #2 includes low SMI and low SMD (binary predictors) using the cutoffs per Martin et al.

^c^Multivariable model #3 includes low SMI and low SMD (binary predictors) using the cutoffs per Xiao et al.

Grade ≥3 postoperative complications were not assessed for 5 patients due to missing data up to 30 days after surgery.

**Supplemental Table 6. Sensitivity analysis of the associations between skeletal muscle characteristics and non-home discharge in patients with an available CT scan ≤2 months before surgery (n=375)**

| **Variable** | **Univariate OR (95%CI)** | ***p*** | **Multivariable**  **OR (95%CI)#1^a^** | ***p*** | **Multivariable**  **OR (95%CI)#2^b^** | ***p*** | **Multivariable**  **OR (95%CI)#3^c^** | ***p*** |
| --- | --- | --- | --- | --- | --- | --- | --- | --- |
| SMI (cm^2^/m^2^)  per 5-unit decrease | 1.31 (1.07-1.61) | 0.008 | 1.04 (0.82-1.30) | 0.77 | Not included |  | Not included |  |
| SMD (HU)  per 5-unit decrease | 1.62 (1.32-1.98) | <0.001 | 1.38 (1.08-1.78) | 0.011 | Not included |  | Not included |  |
| Low SMI per Martin | 2.60 (1.20-5.62) | 0.015 | Not included |  | 1.33 (0.55-3.22) | 0.53 | Not included |  |
| Low SMD per Martin | 6.77 (2.31-19.83) | <0.001 | Not included |  | 3.17 (0.99-10.21) | 0.053 | Not included |  |
| Low SMI per Xiao | 2.47 (1.14-5.35) | 0.021 | Not included |  | Not included |  | 1.08 (0.45-2.58) | 0.86 |
| Low SMD per Xiao | 7.10 (2.42-20.83) | <0.001 | Not included |  | Not included |  | 3.27 (0.99-10.73) | 0.051 |

HUs: Hounsfield units; SMD: skeletal muscle density; SMI: skeletal muscle index

Note: Multivariable models are adjusted for age and Charlson Comorbidity Index due to the small number of events (n=30)

^a^Multivariable model #1 includes SMI and SMD as continuous predictors, each per 5-unit decrease

^b^Multivariable model #2 includes low SMI and low SMD (binary predictors) using the cutoffs per Martin et al.

^c^Multivariable model #3 includes low SMI and low SMD (binary predictors) using the cutoffs per Xiao et al.

Discharge location (non-home): n=6 participants were removed from the analysis due to the following reasons: i) n=4 expired prior to discharge, ii) n=1 were discharged/transferred to court/law enforcement, and iii) n=1 left against medical advice.

**Supplemental Table 7. Sensitivity analysis of the univariate associations between skeletal muscle characteristics and 90-day mortality in patients with an available CT scan ≤2 months before surgery (n=381)**

| **Variable** | **Univariate HR (95%CI)** | ***p*** |
| --- | --- | --- |
| SMI (cm^2^/m^2^)  per 5-unit decrease | 1.31 (0.97-1.77) | 0.080 |
| SMD (HU)  per 5-unit decrease | 1.34 (1.03-1.75) | 0.032 |
| Low SMI per Martin | 2.44 (0.78-7.69) | 0.13 |
| Low SMD per Martin | 2.98 (0.81-11.01) | 0.10 |
| Low SMI per Xiao | 2.32 (0.74-7.30) | 0.15 |
| Low SMD per Xiao | 3.08 (0.83-11.38) | 0.091 |

HUs: Hounsfield units; SMD: skeletal muscle density; SMI: skeletal muscle index

**Supplemental Table 8. Sensitivity analysis of the associations between skeletal muscle characteristics and overall mortality in patients with an available CT scan ≤2 months before surgery (n=381)**

| **Variable** | **Univariate HR (95%CI)** | ***p*** | **Multivariable**  **HR (95%CI)#1^a^** | ***p*** | **Multivariable**  **HR (95%CI)#2^b^** | ***p*** | **Multivariable**  **HR (95%CI)#3^c^** | ***p*** |
| --- | --- | --- | --- | --- | --- | --- | --- | --- |
| SMI (cm^2^/m^2^)  per 5-unit decrease | 1.15 (1.03-1.29) | 0.012 | 0.99 (0.87-1.15) | 0.97 | Not included |  | Not included |  |
| SMD (HU)  per 5-unit decrease | 1.49 (1.24-1.56) | <0.001 | 1.27 (1.09-1.48) | 0.001 | Not included |  | Not included |  |
| Low SMI per Martin | 2.33 (1.46-3.74) | <0.001 | Not included |  | 1.63 (0.96-2.79) | 0.072 | Not included |  |
| Low SMD per Martin | 3.11 (1.83-5.27) | <0.001 | Not included |  | 1.84 (1.01-3.36) | 0.046 | Not included |  |
| Low SMI per Xiao | 2.01 (1.23-3.22) | 0.004 | Not included |  | Not included |  | 1.15 (0.68-1.95) | 0.61 |
| Low SMD per Xiao | 3.18 (1.87-5.38) | <0.001 | Not included |  | Not included |  | 1.80 (0.98-3.30) | 0.058 |

HUs: Hounsfield units; SMD: skeletal muscle density; SMI: skeletal muscle index

Note: Multivariable models are adjusted for age, sex, Charlson Comorbidity Index, tumor stage, neoadjuvant treatment status, and type of surgery

^a^Multivariable model #1 includes SMI and SMD as continuous predictors, each per 5-unit decrease

^b^Multivariable model #2 includes low SMI and low SMD (binary predictors) using the cutoffs per Martin et al.

^c^Multivariable model #3 includes low SMI and low SMD (binary predictors) using the cutoffs per Xiao et al.
